# Qualitative analysis of COVID-19 experiences with testing and vaccination among people with systemic lupus erythematosus in the USA

**DOI:** 10.1007/s10067-025-07814-0

**Published:** 2025-11-21

**Authors:** Lori Brand Bateman, Allyson Hall, Somaia Khamess, Catanya G. Stager, Maria I. Danila, Candace H. Feldman, David H. Chae

**Affiliations:** 1 Division of General Internal Medicine & Population Science, University of Alabama at Birmingham, Heersink School of Medicine, Birmingham, AL USA; 2 Department of Health Services Administration, University of Alabama at Birmingham School of Medicine, Birmingham, AL USA; 3 Department of Physical Medicine & Rehabilitation, John Hopkins University School of Medicine, Baltimore, MD USA; 4 Division of General Internal Medicine & Population Science, University of Alabama at Birmingham, Heersink School of Medicine, Birmingham, AL USA; 5 Department of Clinical Immunology and Rheumatology, University of Alabama at Birmingham School of Medicine, Birmingham, AL USA; 6 Brigham and Women’s Hospital, Division of Rheumatology, Inflammation, and Immunity, Boston, MA USA; 7 Department of Social, Behavioral, and Population Sciences, Celia Scott Weatherhead School of Public Health and Tropical Medicine, Tulane University, New Orleans, LA USA

**Keywords:** COVID-19, Qualitative methods, Social determinants, Systemic lupus erythematosus

## Abstract

**Introduction:**

People with systemic lupus erythematosus (SLE) have faced unique challenges during the COVID-19 pandemic. This study examines these experiences and identifies barriers and facilitators related to COVID-19 testing and vaccination among people with SLE.

**Methods:**

Participants were 25 SLE patients seeking care in two large medical centers in the US South recruited between October 2022 and April 2023. Fifteen one-on-one interviews were conducted; three were with dyads and one was with a group of four people. Sessions were recorded using Zoom Video Communications, which were transcribed and analyzed according to the guidelines of thematic analysis with NVivo 12.

**Results:**

Emerging themes were examined using the social-ecological framework and organized around the impact of COVID-19 and barriers and facilitators of testing and vaccination. Factors that facilitated testing included availability and convenience of home testing; barriers were unclear directions for home tests and lack of accuracy of tests, and lack of clarity about when to get tested. Fear of side effects was cited as a major barrier to vaccination. Healthcare communication about vaccine benefits, particularly in the context of being immunocompromised, were motivating factors.

**Conclusion:**

Interventions may be implemented at multiple levels to address barriers to COVID-19 testing and vaccination among SLE patients. Clear and comprehensive guidelines for those with SLE, as well as efforts to enhance structural competency among providers, including a greater understanding of COVID-19 experiences in the context of SLE, may support protective behaviors in this patient population.

**Table:** 

**Key Points** • *Accurate and up-to-date information from healthcare providers and other trusted sources that is tailored for SLE patients can facilitate testing and vaccination in response to novel infectious disease.* • *Many SLE patients displayed resilience and drew from sources of support in response to unique challenges during the COVID-19 pandemic.*

Systemic lupus erythematosus (SLE) is a chronic autoimmune disease characterized by diverse symptoms, affecting multiple organs and biological systems including the skin, central nervous system, kidneys, and musculoskeletal system [1]. COVID-19 is considered to pose a significant health threat to those with SLE, an autoimmune rheumatic disease associated with the highest risk for infections and related morbidity [2, 3]. Some research suggests that those with SLE are more susceptible to severe COVID-19 consequences, including infection-related complications and hospitalization [4, 5]. The medical and scientific community recommends COVID-19 testing and vaccination as strategies to reduce personal risk of infection as well as transmission to others; testing also helps to identify cases to be referred for treatment to reduce the risk of severe disease. Although evidence suggests that people with SLE are able to safely receive the COVID-19 vaccine, there remain community concerns regarding adverse interactions with medications used to treat SLE, and side effects such as SLE flares and severe infections among patients taking immunosuppressive drugs; and potentially reduced vaccination efficacy [6, 7]. However, the Centers for Disease Control and Prevention (CDC) and the American College of Rheumatology (ACR) indicate that the benefits of COVID-19 vaccination outweigh any potential risks, including among those who are immunocompromised [8].

Social determinants of health have been shown to impact health outcomes among people with SLE; for example, via exposure to health-damaging factors and access to health-promoting resources [9, 10]. Factors associated with gender and race are particularly important to consider in the context of SLE given its disproportionate impact among Black/African American women. Women represent nine out of ten cases and Black/African American women are three times more likely to be diagnosed with SLE compared to all other groups [10, 11]. People with SLE, in addition to facing challenges associated with managing a chronic disease, have also been found to face co-occurring psychosocial demands that may result in greater vulnerability to COVID-19 by compromising self-care and engagement in health-protective behaviors. Black/African Americans in general may have a greater risk of COVID-19 infection and severity due to several reasons, including greater exposure through workplaces (essential occupations), differential healthcare [12, 13], and lower access to testing and vaccination compared to Whites [14]. Black/African American women may be at particular risk for psychosocial strains, including bereavement, heightened multiple role responsibility, competing demands, financial insecurity, and racism-related stressors including racial discrimination [15–17]. These patterns are in part due to environmental and other structural issues tied to racial residential segregation and poverty concentration, greater socioeconomic disadvantage, and lack of health insurance [18–20],

Informational support is critical for enhancing COVID-19 testing and vaccine competency [21, 22]. However, isolation during the COVID-19 pandemic resulted in important informational support channels being disrupted [23]. Furthermore, evolving vaccine and testing recommendations coupled with concerns and misinformation about the side effects and effectiveness of the vaccine made decision-making around COVID-19 difficult [24]. Additionally, existing issues around access to healthcare were magnified due to the prioritization of COVID-19 cases and stay-at-home recommendations resulting in many people with SLE reporting concerns about low provider availability and appointment cancellations [5]. Many people living with SLE also avoided in-person medical visits due to fear of contracting COVID-19 in clinical settings [25]. There were also concerns about shortages in medications used to treat SLE, specifically hydroxychloroquine, which was proposed as a treatment for COVID-19 [26]. Concerns about drug shortages may have resulted in non-adherence to medication regimens among some SLE patients [25]. These stressors resulted in major mental health tolls in this population [27]. Compared to the general population, people living with SLE have been found to be at greater risk of anxiety and depression, as well as sleep disorders during the COVID-19 pandemic [28, 29].

Infectious disease epidemiologists suggest that COVID-19 will remain present for the foreseeable future. For instance, the CDC estimated that between October 2023 and April 2024 there were roughly 41,000 COVID-related hospitalizations [30]. During the 2024–2025 respiratory virus season, between 2.4 million and 4.4 million people have had COVID-19 [21]. Identifying strategies that facilitate protective behaviors such as testing and vaccination, particularly among those at greater risk for severe disease, remains a public health concern.

Studies examining the experiences of people with SLE during the COVID-19 pandemic have looked at public health communications and patient behavior [31], reasons for vaccine hesitancy for adults with immune-mediated inflammatory diseases [32], and interventions post-pandemic to improve health outcomes [33]. While there are some qualitative studies that explore the perspectives of those with SLE, research examining barriers and facilitators to testing and vaccination using a social-ecological lens is lacking [10, 34]. Insights garnered from those in the US South, a region with higher SLE prevalence rates, compared to the rest of the country, may be particularly relevant [35, 36].

The purpose of this study is to qualitatively examine COVID-19 related experiences among people with SLE, recruited from two large medical centers in the Southeast, particularly factors associated with (1) testing and (2) vaccination (which may be distinct) through a social–ecological framework.

## Methods

### Participants and recruitment

Participants were recruited between October 2022 and April 2023 during clinic visits and through self-referral from posted advertisements and direct recruitment during patient visits at two large academic medical centers in the US South. Recruitment materials solicited participants for a study about the experiences of people living with SLE during the COVID-19 pandemic. We intended to conduct focus groups and one-on-one interviews based on vaccination status, race, and gender which we hypothesized were important characteristics associated with testing and vaccination; however, due to scheduling constraints and difficulties identifying common times that participants were available, the majority were interviewed individually. Groups and individual interviews were conducted using Zoom Video Communications, Inc. Interviewers/moderators underwent training to standardize methods, including those around probing. We used a purposive sampling approach [37], and in determining our sample size, we aimed for data saturation, defined as the point where no new codes arise from qualitative data [38], which we estimated to be from 20 to 25 participants.

There were 25 participants—24 women (17 Black/African American, six White, and one multi-racial Asian/White), and one White man. Participant sociodemographic characteristics are shown in Table 1. Three interview sessions were conducted with pairs, and of these, two were of Black/African American women who had been vaccinated, and one consisted of White vaccinated participants with different genders. One interview with a group of four vaccinated Black/African American women was conducted. Individual interviews were conducted with 15 participants, three of whom never received a COVID-19 vaccine dose. Individual interviews averaged 30 min in duration, and participants received a $20 incentive. Those interviewed in groups averaged 90 min and participants received a $40 incentive. The protocol was approved by the Institutional Review Board of the University of Alabama at Birmingham.

**Table 1:** Sociodemographic characteristics of interview participants (*N* = 25)

Sociodemographic characteristic	*n* (%)
Race, *n* (%)	
Black	17 (68.0%)
White	7 (28.0%)
Multiracial	1 (4%)
Gender	
Women	24 (96.0%)
Men	1 (4.0%)
Age, *n* (%)	
18–34 years	4 (16.7%)
35–49 years	8 (33.3%)
50–64 years	9 (37.5%)
65 years or older	3 (12.5%)
Years since diagnosis, *n* (%)	
< 10 years or more	6 (26.1%)
10–14.9 years or more	7 (30.4%)
15–19.9 years or more	4 (17.4%)
20 years or more	6 (26.1%)
Education, *n* (%)	
High school or less	2 (8.3%)
Some college	13 (54.2%)
Bachelor’s degree	4 (16.7%)
Graduate/professional degree	5 (20.8%)
Household income, *n* (%)	
< $25,000	6 (26.1%)
$25,000–$49,999	3 (13.0%)
$50,000–$74,999	4 (17.4%)
$75,000–$99,999	6 (26.1%)
≥ $100,000	4 (17.4%)
Recruitment site, *n* (%)	
Alabama	20 (83.3%)
Louisiana	4 (16.7%)
COVID-19 vaccination status, *n* (%)	
Any	22 (88.0%)
None	3 (12.0%)

Total *n* may not equal 25 due to missing data

### Interview and focus group protocol

We aimed to achieve saturation of themes related to barriers and facilitators of COVID-19 testing and vaccination, and to identify strategies that would be most relevant or effective in promoting these behaviors. The semi-structured interview guide included two sub-sections: (1) for those who had not received any COVID-19 vaccinations, we explored the reasons for not being vaccinated, what would motivate them to get tested and vaccinated, including specific strategies that would be the most relevant or effective; (2) for those who had already received at least one dose of the COVID-19 vaccine, we asked them about the reasons they decided to be vaccinated and what strategies might be effective in encouraging others who have not yet been vaccinated to do so.

#### Analysis

Transcripts were anonymized and the final dataset was stripped of identifiers. Data were analyzed using NVivo 12, and a team of three analysts worked together to code and analyze the transcripts according to the guidelines of Thematic Analysis [39]. Two of the analysts (LB and AGH) read through six transcripts independently (four interviews and two groups) to develop a broad coding structure, then worked with a third coder (SK) to reconcile their coding structures into a single codebook. After the coding agreement was established, transcripts were coded by one analyst (SK) and coding was substantiated by another (LB).

## Results

### Conceptual framework

Themes were conceptualized using a socio-ecological framework and organized around concepts of the impact of COVID-19, and barriers and facilitators of COVID-19 testing and vaccination. According to ecological systems theories [40], the individual is situated in overlapping social and cultural systems, and is embedded within macrosystems including interpersonal relationships, structural community constructs, and society. As a systems approach, the social ecological model outlines outcomes as structures and processes that interact with one another dynamically [41]. A summary of themes identified at the individual, interpersonal, and structural levels is presented in Table 2.

**Table 2:** Themes on facilitators and barriers to COVID-19 testing and vaccination identified from interviews and focus groups using the social ecological model (*N* = 25)

	COVID-19 testing		
	Individual	Interpersonal	Structural
Facilitators	• Suspecting exposure to COVID-19 • Trust in home testing • Work-related testing requirements	• Healthcare provider recommendations • Requiring others to get tested out of fear of exposure • Fear of isolation and loneliness	• Convenience of home testing
Barriers	• Mistrust of test accuracy • Uncertainty about when to get tested		• Unclear home testing instructions • Scarcity of home testing kits
	COVID-19 vaccination		
	Individual	Interpersonal	Structural
Facilitators	• Seeking protection from COVID-19 • Seeking protection for family • Seeing no side effect on other who got vaccinated	• Messages about vaccination from doctors • Encouragement from others	• Work requirement
Barriers	• Fear of vaccine-related effects on lupus patients • Adopted attitude against all vaccines	• Political affiliation	• Mixed messages
Strategies to improve vaccination uptake	• Importance of healthcare professionals delivering a persuasive message • Benefit of establishing lupus peer navigators • Creating online support groups		

### COVID-19 testing

Overall, participants considered COVID-19 testing to be a key part of their health management and they engaged in frequent testing. There was a reliance on at-home testing kits, and many participants had these readily available.

#### Facilitators of COVID-19 testing

*Individual-level* factors that motivated COVID-19 testing included exposure to COVID-19, availability of home testing, and COVID-19 testing as work requirements. Many participants mentioned that they tested when they experienced any new symptoms that deviated from their usual baseline that could potentially be related to COVID-19, such as unexplained fatigue, fever, or coughing, and considered home testing as a primary way to rule out infection. Some reported testing even when exposure or symptoms were considered minor. One vaccinated Black/African American woman described the difficulty of determining if new symptoms were usual or COVID-19-related:

…Because we do know that lupus, of course, mimics respiratory, symptoms at times. You know, sometimes you feel like you got the flu, you know? And but it, what it is, is your lupus, so if you feel like something is not right and it’s something that doesn’t seem like it’s normal for your normal flare-ups or your normal lupus day.

They also opted for testing when they had been in contact with a symptomatic individual or someone who recently tested positive. Others revealed that COVID-19 testing was mandated by their workplace, academic institution, or healthcare facility, and compliance with regular testing was necessary to access these locations.

*Interpersonal-level* factors included being advised by healthcare providers to get tested whenever they experience new symptoms. To protect themselves, patients described that they encouraged their friends and family to get tested before face-to-face interactions, although many avoided interactions altogether out of fear. Fear was an often-repeated word during the study, related to the unpredictability of COVID-19 infection in SLE patients. Participants were concerned about the potential of severe symptoms because of their immunocompromised state. They also feared isolation and loneliness due to limited social interactions. Even as society seemed to move on from the pandemic, fear remained for people with lupus. For example, one participant stated:

…a lot of people assume that COVID is over because the media has downplayed it so much and it’snot being covered as much, but COVID is still very much active and alive.

*Structural-level* factors that were cited included the convenience of mailed home tests which allowed them to perform the test themselves and receive results within 15 min. Some participants also appreciated COVID-19 testing being free of charge.

#### Barriers to COVID-19 Testing

Barriers to COVID-19 testing at *the* *individual level* included mistrust of home testing accuracy and uncertainty about when to get tested. Many participants mentioned receiving different test results at the doctor’s office or urgent care, compared to home tests, which led them to mistrust results from home test kits. Also, the similarities between COVID-19 and lupus symptoms created confusion. Participants sometimes got tested when in doubt, but occasionally missed the testing timeframe, only later to discover they were previously infected with COVID-19.

*Structural-level* barriers, including unclear instructions for home testing kits, exist. The presence of multiple brands, each with its own set of instructions, was confusing and contributed to a lack of trust in home test results. Some patients addressed this issue by searching for illustrative videos online and doing more than one test. A few participants encountered difficulties due to the unavailability of home testing kits at stores or their high cost. Additionally, tests delivered by mail often expired before they could be used. For PCR testing, a few participants used terms like “horrible,” “painful,” and “uncomfortable” to describe their experiences with it. A vaccinated Black/African American woman described her struggle with home tests:

...Well, I’m glad they had like three of them in the box when you got it because the first one I was like, ohh Lord, I didn’t think I missed the step. So the first one I messed up but the second one I took. And it showed that it was positive. So when I took it again with the third test, it was negative.

Participants suggested that healthcare providers instructing them on how to perform the home test could help them do it correctly.

### COVID-19 vaccination

Participants’ experiences with vaccinations were described as straightforward, with easy access to vaccination sites and boosters. Many participants consulted with healthcare professionals before getting vaccinated. Several participants mentioned mild side effects, such as sore arms, fever, body aches, and nausea.

#### Facilitators of COVID-19 vaccination

Facilitators at the *individual level* included seeking protection from COVID-19 and believing in the safety of vaccinations. Most participants emphasized that vaccination is essential to reduce the risk of severe illness, hospitalization, the need for a ventilator, long-term complications, or even death, particularly due to their pre-existing health conditions and compromised immune systems. They expressed a preference for being overly cautious rather than taking any risks. They believed that once the virus is contracted, it may be too late to avoid the regret of not getting vaccinated. They were also motivated by the desire to protect their family members. Finally, seeing no side effects in others who got vaccinated, supported by testimonies from other vaccinated lupus patients, played a convincing role. A vaccinated White woman expressed her reasons for seeking vaccination:

...if it was going to keep me from being on the ventilator or losing my life and my family not having me here... that was one of the main things... and a lot of people said that it was going to, it was going to make it worse if you didn’thave the vaccine.

*Interpersonal-level* factors included advice from their doctors, such as rheumatologists and primary care physicians, which helped them feel confident about getting vaccinated and boosted. Some participants appreciated that doctors did not exert pressure on them to get vaccinated, but explained the benefits of vaccination and provided recommendations that helped them make their own decisions. For example, one participant explained her doctors’ influence on her decision to get vaccinated:

Neither one of them was pushing it, so I don’t feel like they influenced my decision at all. They didn’t sway me one way or another, and I appreciated that. They allowed me to make my own decision so that, I was grateful for that. (vaccinated Black/African American woman)

In addition, many participants highlighted the influence of people in their social circles, such as fellow lupus patients, close friends, and coworkers, in their vaccine decision-making. Participants engaged in conversations, reached out, and inquired about others’ experiences with vaccines, particularly regarding side effects. Such personal testimonials and interactions, both in person and through social media, played a crucial role in reassuring them and aiding their decision-making process. One patient mentioned:

… having a friend in public health and her like, watching out for me like she would be like, ‘Oh, you can get the vaccine now. You know, you’rein the particular group,’ was….actually really helped too.

A structural-level factor that some participants reported, particularly those working in healthcare settings or on college campuses, was employers’ requirements to get vaccinated.

*Individual-level* barriers to COVID-19 vaccination included fear of vaccine-related side effects and having an attitude against all vaccines. Many participants shared their concerns which included the speed at which the vaccines were developed and the unknown long-term effects and systemic impact of the vaccine, especially in the context of having a preexisting health condition and being immunocompromised. Participants also expressed uncertainty about the vaccine’s possible interaction with existing medications, and adverse effects of vaccines observed in others such as blood clots. A vaccinated Black/African American woman summarized her concerns:

...Because they’re scared you don’t know what’s in it. They don’t tell you everything that’s in it. And because you don’t know how your body is going to respond. I mean, darn, we don’t know how we’re going to respond to the medications as lupus that we are given.

A few participants, all of whom were not vaccinated, mentioned negative past experiences with vaccines, such as catching the flu after influenza vaccination or experiencing allergic reactions to certain vaccine components. Some mentioned cultural beliefs that discouraged vaccination. For example, an unvaccinated White woman stated:

Well, I usually don’t get vaccines like the flu vaccine and live vaccines basically. I usually end up with the flu if I get the flu shot. So I was afraid that if I got the COVID shot that I would end up with COVID. So I just really, just overprotecting myself with, you know, a mask and hand sanitizer and all.

Further, some people mentioned feeling pressured. Others were discouraged from getting the vaccine based on their political affiliations.

*Structural-level* barriers included mixed information they received from various sources related to the vaccine, and reports of severe reactions to the vaccine such as blindness, skin problems, and even brain aneurysms. Perceived discrepancies between what the CDC recommended and what was reported in the news left some participants feeling confused and hesitant.

### Strategies to improve vaccination uptake

Participants highlighted the need to provide comprehensive information regarding COVID-19 vaccines, such as clarifying the safety profile of vaccines, and how vaccines function for those immunocompromised, potential interactions with their medications, vaccine components, and detailed data on side effects experienced by lupus patients. Such information could be distributed at hospitals and through healthcare provider websites and social media platforms.

Additionally, there was agreement on the importance of healthcare professionals delivering persuasive messages that highlighted the benefits of vaccination not only for lupus patients but also for their immediate circle of family and friends. Participants also suggested the benefits of establishing lupus peer navigators or educational navigators who can address questions specific to lupus patients and their vaccination experiences. One participant mentioned how important testimonies of other patients with lupus were:

… knowing that someone with lupus has... been through the situation and seeing the outcome of being vaccinated. What’s the benefit of being vaccinated…So testimony is a big part of helping a lupus patient get vaccinated.

Creating online support groups was suggested to offer additional assistance and information-sharing opportunities.

## Discussion

This study reports on the lived experiences of people with SLE related to COVID-19—the impact that it has had on their lives, and facilitators and barriers to testing and vaccination. Utilizing a socioecological framework, participant responses regarding facilitators and barriers were identified at the individual, interpersonal/relationship, and structural levels. Overall findings at the individual level indicate that COVID-19 had a tremendous impact on SLE patients. However, despite the unique challenges they experienced, many showed signs of resilience and adaptation to the evolving landscape and took deliberate steps to protect their health.

In the current study, participants with SLE reported exhaustive preventive measures and daily lifestyle changes, as well as fear, reflecting the wide-ranging impact of COVID-19. The continued use of extensive preventive measures among SLE patients contrasted with the increasing relaxation of preventative measures in the general population as the pandemic evolved [4, 42]. Individuals with chronic diseases, such as SLE, tend to show greater caution and engage in preventive measures more than the average individual [7]. Additionally, those with SLE require very specific information regarding how COVID-19 vaccination could affect their health [43].

Our results align with previous COVID-19 studies that demonstrate that patients with SLE are at greater risk of negative mental health outcomes such as anxiety and depression [39]. Studies with clinically vulnerable patients have found that high levels of anxiety and increased feelings of fear were associated with lifestyle changes, including shielding behaviors [3]. Isolation, decreased access to healthcare, and increased health risk have been themes reported in other COVID-19 studies of SLE patients [28].

Participants cited prior negative experiences in healthcare, lack of trust in home testing accuracy, and uncertainty about when to get tested as barriers to testing and vaccination. Psychosocial stressors experienced by people with SLE can impact their decision-making processes regarding COVID-19 prevention behaviors. Structural-level factors such as unclear instructions for home testing kits, variability in brands, and difficulties in accessing reliable testing options constituted environmental barriers for those with SLE. Challenges associated with managing COVID-19 difficulties may further exacerbate the psychological burden on SLE patients [39], affecting their ability to engage in COVID-19 prevention measures.

Although the racial and gender composition of participants in our study mirrors those of the SLE population, our findings may not be generalizable to all groups, particularly those living in other geographic areas or those seen outside academic medical centers who may face different barriers, particularly at the structural level. Another caveat to our study is that findings may not be generalizable to men. The one man in our study highlighted that SLE is often considered a woman’s disease with very little consideration of men’s perspectives. Additional research should be conducted to examine the unique experiences of men with SLE in the context of COVID-19. In addition, we did not explicitly examine any differences by race given the relatively small sample size of participants, and because race-specific factors were not explicitly mentioned by participants. The overarching goal of this study was to inventory the barriers and facilitators to COVID-19 testing and vaccination expressed by people with SLE broadly and to achieve saturation of themes. With that said, however, it is possible that themes reported may vary in frequency, degree, and salience along sociodemographic lines, including not only by gender and race, but also by socioeconomic status and age, for example, among others. Future research may examine experiences at the intersection of SLE and social identities when investigating COVID-19 behaviors. In addition, findings from this study are specifically for health behaviors for COVID-19; they may not apply to testing or vaccination for other diseases (e.g., influenza) or other emerging therapies for disease management or treatment of SLE. Although we did not detect evidence of systematic differences in information shared in one-on-one interviews versus in a group format, we cannot discount this possibility.

Insights gained from this study have important implications for public health strategies aimed at mitigating COVID-19 and potentially other novel infectious diseases, particularly among more susceptible groups. For instance, workplaces may accommodate the unique needs of those who are immunocompromised and adjust policies to support a healthy employee environment. Consistent messaging from healthcare providers and de-politicization of testing and vaccination may also help promote these protective behaviors. Additional studies may examine the most effective methods of informing healthcare providers about emerging new vaccines and therapies and keeping them continuously updated as information evolves.

Results from this study can inform the development of targeted interventions and support programs to improve adherence to recommended guidelines (in addition to providing guidelines that are easy to understand) among people with SLE. Informing patients on common SLE and COVID-19 symptoms and guiding them in recommended preventative practices particularly by healthcare providers may also be an effective strategy. Our participants highlighted the need for comprehensive education for those with SLE experiencing COVID-19, and a decision support tool may be created that incorporates specific vaccine decisions for those with SLE. Developing community engagement efforts can facilitate the provision of accurate information, such as engaging patient navigators and online or in-person support groups. Adopting these approaches may help mitigate the adverse impact that COVID-19 has had on the physical and mental health of people with SLE.

## Conclusion

The current study explores perspectives on COVID-19 testing and vaccination among a sample of SLE patients. We identified unique issues expressed in the context of this autoimmune disease and identified barriers and facilitators to testing and vaccination at multiple levels. By engaging people directly about their personal experiences during the pandemic, public health can more effectively identify relevant points of intervention, provide recommendations, and promote feasible solutions to enhance health management and promote the well-being of people with SLE.

## Data Availability

The data that support the findings of this study are available from the corresponding author upon reasonable request.
